# Een veerkrachtige publieke gezondheid in 2030: #hoedan?

**DOI:** 10.1007/s12508-022-00364-9

**Published:** 2022-10-28

**Authors:** Luc Hagenaars, Wilma Waterlander, Karen den Hertog, Karien Stronks

**Affiliations:** 1 Department of Public and Occupational Health, Amsterdam Public Health research institute, Amsterdam UMC locatie AMC, Amsterdam, Nederland; 2 Afdeling Gezond Leven, Gemeente Amsterdam, GGD Amsterdam-Amstelland, Amsterdam, Nederland

**Keywords:** systeemdenken, complex-adaptieve systemen, beleidsproces, Systems thinking, Complex-adaptive systems, Policy process

## Abstract

Bij gamechangers in public health wordt vaak automatisch gedacht aan specifieke interventies of uitvindingen. Vroeger was dat het riool, later werden het vaccinaties en tegenwoordig gelden overspannen verwachtingen van leefstijlinterventies en (opnieuw) vaccinaties. Dit denken in termen van *silver bullets* heeft ons veel gebracht, maar loopt vast in de complexiteit van de huidige uitdagingen van de publieke gezondheid. In dit essay schetsen we het erkennen van en handelen vanuit die complexiteit als gamechanger. We beschrijven wat het denken in termen van complexiteit betekent voor de volksgezondheid, en wat dit vraagt van de publieke gezondheidszorg.

## Abstract (en)

Game changers in public health are traditionally seen as specific in(ter)ventions, but contemporary public health challenges warrant acknowledging complexity instead. Our game changer is a compass to deal with this complexity. In our vision of 2030, system beliefs, goals, structures and events line up to create a society that balances health, climate, social cohesion and economy. To reach this desired system, a resilient public health sector actively interacts with public discourse, political windows of opportunity are seized for institutionalizing health for all policies, and research is intertwined with the policy process, without the merging of the two.

## Winnaar van de TSG-Challenge

In 2022 bestaat *TSG – Tijdschrift voor Gezondheidswetenschappen* honderd jaar. Voor die gelegenheid heeft *TSG* een schrijfwedstrijd uitgeschreven: de TSG-Challenge. We vroegen het veld om een opiniërend onderbouwd artikel te schrijven over dé gamechanger voor de volksgezondheid in 2030. Uit de inzendingen heeft de jury één winnaar en twee eervolle vermeldingen gekozen.

Dit artikel is het winnende artikel. De jury zegt over deze bijdrage: ‘Het beleidsconcept van complexiteit doet naar het oordeel van de jury recht aan de samenhang van de problematiek van volksgezondheid en is wetenschappelijk goed onderbouwd. De brede samenhang tussen gezondheid, economie, duurzaamheid en andere actuele grote vraagstukken wordt adequaat opgepakt. Ook looft de jury de aandacht voor de rol van professionals in de verandering. De aanpak neemt veel op de schop en zou daarmee potentieel grote impact kunnen hebben, maar het is wel wat abstract en hier en daar te zwart-wit: de gewenste verandering in 2022 wordt iets te mooi neergezet en is nog weinig concreet. Dat vraagt zeker verdere uitwerking.’

Lees het hele juryrapport hier: link 10.1007/s12508-022-00360-z.

## De publieke gezondheid in 2030

Het is januari 2030. De afgelopen jaren zijn grote stappen gemaakt in het vinden van structurele oplossingen voor de grote gezondheidsvraagstukken van deze tijd. Gamechanger bleek het erkennen van complexiteit. We raakten verlost van het denken in determinanten, individueel gedrag en leefstijlinterventies. In plaats daarvan zijn we de volksgezondheid, net als een gezond milieu, sociale samenhang en economische voorspoed, gaan zien als de uitkomst van een systeem waarin een breed scala aan elementen met elkaar interacteert [1].

Er is bovendien een balans gevonden tussen gezondheid, sociale samenhang, milieu en economie. Tot het begin van de 21ste eeuw werd de samenleving gestuurd vanuit één systeemdoel: economische groei. Het was vrijwel onmogelijk om impact te hebben op doelen die niet direct bijdroegen aan groei of daar tegenin gingen. Een voorbeeld. In 2022 was 80 procent van onze voedselomgeving ongezond. De verkoop van junkfood was winstgevender dan die van gezonde voeding. Hierdoor maakte een klein aantal bedrijven grote winsten, waardoor grote marketingbudgetten en politieke lobby’s de status quo in stand hielden. Totdat de samenleving tegen dit systeem opstond. De winstgevendheid van ongezonde en milieubelastende voeding kwam op de helling door regulering van junkfood in de openbare omgeving. Maar daar bleef het niet bij. Anno 2030 zijn ook de volgende gezondheidsbeschermende maatregelen zó vanzelfsprekend dat we ons er nauwelijks bewust van zijn: invoering van maximumsnelheid tot 80 km/uur op rondwegen, toetsing van gemeentelijk beleid op duurzaamheid en leefbaarheid, de keuze voor bestaanszekerheid als leidend principe in de sociale zekerheid.

Het geschetste toekomstbeeld leek in 2022 utopisch, maar was dat zeker niet. Complex-adaptieve systemen zoals de volksgezondheid, waarvan de onderling verbonden componenten zich voortdurend aanpassen aan veranderingen in de omgeving, zijn immers per definitie in beweging. Zo zijn we in staat gebleken het slavernijsysteem af te schaffen, iets wat ooit praktisch onmogelijk werd geacht. Beweging naar een nieuw systeem vergt geen onmenselijke krachtinspanningen, maar vraagt vooral dat we denken en handelen vanuit het perspectief van complexe systemen [2].

De verschuiving in het denken over de volksgezondheid richting het perspectief van complexe systemen startte zo halverwege het tweede decennium. Dit aarzelende begin leidde niet onmiddellijk tot een effectievere praktijk, getuige de leefstijlgerichte benadering van het eerste Preventieakkoord in 2018 en het logische gebrek aan gezondheidsimpact ervan. Maar de visie dat gezondheid een product van de maatschappij als geheel is, werd wel steeds breder gedeeld [3], mede omdat de COVID-19-crisis zo duidelijk de ongelijke gezondheidskansen blootlegde.

Het kompas voor de omgang met complexiteit werd gevonden in de vier interacterende niveaus uit het systeemdenken van Meadows [2]. In toenemend belang gaat het om de feitelijk genomen beleidsmaatregelen zoals hierboven geschetst, de infrastructuur waarbinnen deze maatregelen tot stand komen, en de doelen en overtuigingen die daar onder liggen. We beschrijven hoe inzetten op overtuigingen, beleidsdoelen en de infrastructuur anno 2030 tot een veerkrachtige publieke gezondheid heeft geleid. Met veerkrachtig bedoelen we een publieke gezondheid die gebaseerd is op een goed inzicht in de werking van het complexe systeem dat de volksgezondheid vormt, en die strategisch handelt in lijn met de systeemveranderingen die nodig zijn om de volksgezondheid te verbeteren. Tabel 1 vat de leidende principes voor en na deze transitie samen.

**Table 1:** 

2022	2030
*niveau 1 – overtuigingen*	
leefstijl	leefomgeving
determinanten	onderliggende mechanismen
gezondheidsinterventies	veranderingen aanbrengen in het systeem dat (on)gezondheid produceert
gezondheidsbevordering	gezondheidsbescherming
eigen verantwoordelijkheid	collectieve verantwoordelijkheid
gezonde keuze makkelijke keuze	gezonde omgeving
selectieve preventie	proportioneel universeel beleid, dat wil zeggen beleid dat voor iedereen geldt, maar dat proportioneel is naar mate van achterstand, waardoor degenen met een grotere gezondheidsachterstand meer baten ondervinden
belangen van economie en volksgezondheid conflicteren	goede gezondheid is een voorwaarde voor een gezonde economie en vice versa
*niveau 2 – beleidsdoelen*	
intersectoraal beleid	integrale doelen, aangevuld met gefragmenteerd beleid
health in all policies	health for all policies
zoeken naar mogelijkheden binnen de bestaande beleidsdoelstellingen	gezondheidsdoelen verweven met doelen van relevante sectoren
*niveau 3 – infrastructuur*	
één probleemeigenaar	meerdere probleemeigenaren
gezondheidsmonitor	brede welvaartsmonitor
implementatie van wetenschappelijke kennis in het beleid	onderzoekers en beleidsmakers zijn partners bij de ontwikkeling van maatregelen
onderzoeksmethode om te weten wat werkt (attributie)	onderzoeksmethoden om te snappen wat bijdraagt aan (contributie)
andere sectoren adviseren hoe ze aan gezondheid kunnen werken	andere sectoren helpen met hun eigen (gezondheids)doelen
*niveau 4 – maatregelen (voorbeelden)*	
leefstijlinterventies (bijvoorbeeld Gecombineerde Leefstijl Interventies of kookcursussen)	aangrijpingspunten in de fysieke, sociale, economische en digitale leefomgeving (bijvoorbeeld beloopbaarheid van voorzieningen, de sociale norm rond zonnebaden, gezonde voedselzekerheid, en een verbod op crypto’s en gokken)

## Niveau 1 – Overtuigingen in 2030

Welke overtuigingen bleken cruciaal in het dragen van de systeemveranderingen? We noemen er twee: hoe gezondheid wordt geconceptualiseerd en hoe we gezondheid wegen ten opzichte van andere maatschappelijke doelen.

### Conceptualisatie van gezondheid

Terwijl gezondheid lange tijd werd gezien als afhankelijk van toeval (genetica) en individueel gedrag, liet de coronacrisis zien dat dit feitelijk onjuist is. ‘Ziektelast bij mensen wordt grotendeels door omgevingsfactoren bepaald’, zo schreef de Gezondheidsraad in 2022 [4]. Het denken van beleidsmakers en politici is veranderd. Verwijzingen naar de sociale en fysieke context van gedragingen zijn niet langer uitstapjes binnen de conceptualisatie van gezondheid als een individueel goed, maar beklijven, omdat ze onderdeel uitmaken van het beeld dat burgers en machtshebbers van gezondheid hebben.

De coronacrisis zette de schijnwerpers op deze denkomslag, waardoor steeds meer mensen betrokken raakten bij de al langer lopende discussie over gelijke kansen. Juist succesvolle (en doorgaans invloedrijke) mensen gingen er lang vanuit dat hun succes het gevolg was van hun persoonlijke competenties. Zij werden door de toenemende kansenongelijkheid echter steeds meer geconfronteerd met wat minder succesvolle mensen allang wisten, namelijk dat mensen in lagere posities niet voor ongezondheid hebben gekozen. Het besef dat gezondheid geen keuze is kreeg extra wind in de zeilen toen in de jaren twintig, als gevolg van klimaatverandering, ook in Nederland extreem weer steeds vaker de volksgezondheid bedreigde.

### Gezondheid ten opzichte van andere maatschappelijke doelen

De hierboven geschetste veranderingen in overtuigingen kwamen niet zonder slag of stoot tot stand. Dat de publieke gezondheidszorg de aandacht voor preventie tijdens de COVID-crisis in eerste instantie maar beperkt wist aan te grijpen voor effectiever preventiebeleid, noopte de sector tot zelfreflectie. Evaluatie leerde dat de publieke gezondheid beter had kunnen aansluiten bij de overtuigingen van de politieke meerderheid. Deze hekelde de idee dat de pandemie de economie in gevaar bracht. Publieke gezondheidsexperts positioneren gezondheid eind jaren twintig daarom als voorwaarde voor groei. Het werd steeds duidelijker dat er geen algemene tegenstelling tussen volksgezondheid en economie is, maar dat gezondheidsmaatregelen kunnen conflicteren met economische deelbelangen.

Deze overtuigingen vielen in vruchtbare grond, als gevolg van een groeiend besef dat specifieke bedrijven de volksgezondheid – en daarmee de economische groei – beschadigen. Het werd duidelijk dat de *devil shift*, waarbij slechte eigenschappen, slechte doelen en slecht gedrag van een belanghebbende worden aangedikt [5], helpt om doorbraken te forceren. Werd deze tactiek eerder met succes tegen de tabaksindustrie ingezet, anno 2030 wordt dit succes tegen de voedings- en game-industrie herhaald.

## Niveau 2 – Beleidsdoelen in 2030

Hoe zijn beleidsdoelen mee veranderd met de verschuivingen in overtuigingen? De *Punctuated Equilibrium Theory* bleek een goede basis voor het nastreven van gezondheidsdoelen [5]. Deze theorie stelt dat politici niet in staat zijn om aan alle problemen tegelijkertijd voldoende aandacht te schenken. Beleidssilo’s claimen eigenaarschap op een thema en hebben een belang bij de status quo. Intersectorale samenwerking lukt alleen als het probleemeigenaarschap niet in gevaar komt. Maar wanneer het maatschappelijk beeld van een probleem kantelt, geldt een andere dynamiek. Politieke kopstukken worden dan (tijdelijk) actief probleemeigenaar, om beleid integraal en fundamenteel te veranderen.

### Integrale wettelijke gezondheidsdoelen in 2030

Gezondheidsprofessionals grepen in de jaren twintig de kantelende overtuigingen met beide handen aan om te pleiten voor wettelijke streefwaarden voor de volksgezondheid [6]. Deze streefwaarden institutionaliseerden blijvende aandacht voor gezondheid ten opzichte van andere belangen. Het was even zoeken naar een gezondheidsequivalent voor CO_2_-uitstoot, zodanig geformuleerd dat politici ervoor verantwoordelijk gehouden konden worden. Epidemiologische inzet om dieperliggende, beïnvloedbare oorzaken van ongezondheid te meten, zoals de dichtheid van junkfood in wijken en een uitgebreide taxonomie van bestaansonzekerheid, leverde niettemin een valide meetinstrumentarium op.

Deze ‘wettelijke doelen voor gelijke gezondheidskansen’ hadden majeure effecten. Omdat ze goed uitgelijnd waren met de klimaat- en brede welvaartsdoelen, ontstond ruimte voor integraal in plaats van intersectoraal beleid. Het verschil is als volgt uit te leggen. Intersectoraal beleid impliceert de betrokkenheid van verschillende publieke sectoren, zoals de zorg, het onderwijs of de sociale zekerheid, via een (her)verdeling van middelen. De sector van de publieke gezondheid heeft in dat proces een coördinerende rol waar het gaat om de volksgezondheid. Gelet op de oorspronkelijke dominantie van interventies ter bevordering van gezond gedrag (zie tab. 1), resulteert intersectoraal beleid vaak in gezondheidsbevordering bij kwetsbare groepen. Dit bleek maar beperkt effectief. Integraal beleid start daarentegen vanuit het vaststellen van doelen, waarna verantwoordelijke sectoren de uitvoering zelf op zich nemen. Betere regulering en dus effectievere gezondheidsbescherming is daarvan het logische gevolg [7]. Vooral beleidssilo’s die de omgeving vormgeven (zoals ruimtelijke ordening, duurzaamheid, economische zaken) bleken hierin bedreven. Nadat de ruimtelijke ordening probleemeigenaar werd van de voedselomgeving, werd bijvoorbeeld snel uitvoerbaar beleid gemaakt van de wens om het aanbod van ongezonde voeding te reduceren.

### Beter gefragmenteerd beleid

Publieke gezondheidsprofessionals creëerden een tweede doorbraak toen ze stelselmatig andere beleidssilo’s hielpen om hun gezondheidsdoelen én de doelen op hun eigen terrein te behalen. Dat veel kinderen zonder ontbijt naar school gingen bleek bijvoorbeeld een van de oorzaken van de verslechtering van lees- en rekenvaardigheden. Stapsgewijs werd schoolontbijt ingevoerd op scholen waar veel kinderen zonder ontbijt naar school gaan.

Met deze constructieve houding heeft de publieke gezondheid definitief afscheid genomen van het eenrichtingsverkeer van het benoemen van volksgezondheidsbelangen naar andere sectoren toe. Health *in* All Policies is vervangen door de term Health *for* All Policies [8], en het blijft niet bij semantiek. De publieke gezondheidszorg heeft *skin in the game* gekregen: het tuigt bijvoorbeeld snel passend onderzoek op om opkomende vraagstukken in relevante sectoren te helpen ontleden. Anno 2030 is er sprake van integraler beleid, en dat komt van twee kanten.

## Niveau 3 – Publieke gezondheidsinfrastructuur in 2030

De verandering van beleidsdoelen ging gepaard met infrastructurele veranderingen. We focussen op twee aspecten: de wijze waarop onderzoek en beleid interacteren en de competenties van professionals.

Nederland kent traditioneel een sterke infrastructuur voor gezondheidsmonitoring. Het besef van de complexiteit van volksgezondheidsproblemen leidde er na de coronacrisis toe dat reguliere gezondheidsmonitoring werd aangevuld met data over achterliggende sociale determinanten. Deze verandering kon snel plaatsvinden, omdat men aansloot bij een al langer lopende ontwikkeling binnen de economie om de brede welvaart in plaats van het BBP centraal te stellen [9].

Die monitoring staat niet op zich, maar is nauw verweven met beleidsontwikkeling. In teams met onderzoekers, beleidsmakers, maatschappelijke organisaties en burgers worden gezamenlijk analyses gemaakt van het complex van onderliggende oorzaken van een opkomend volksgezondheidsprobleem, van de ingangen om dat systeem te veranderen, en van de gevolgen daarvan voor andere maatschappelijke doelen. Er wordt aan de ene kant erkend dat kennis niet beleidsneutraal is en anderzijds dat beleid niet zonder kennis kan. Deze erkenning bleek cruciaal voor daadwerkelijk door kennis geïnformeerd beleid.

Deze structuurwijzigingen konden alleen plaatsvinden doordat de competenties van publieke gezondheidsprofessionals in lijn raakten met het gewenste toekomstbeeld. Onderzoek liet zien dat de ultieme gamechanger van de publieke gezondheid, het riool, te danken was aan de politieke sensitiviteit, de onderzoekscapaciteiten én het streven van hygiënisten om minderbedeelden te helpen [10]. Die laatste twee competenties hoefden enkel gekalibreerd te worden op de complexiteit van de huidige volksgezondheidsproblemen. De politiek-bestuurlijke competenties vroegen overigens meer revitalisatie.

## Breaking nieuws brengt je ineens terug in de realiteit van december 2022

De nieuwe coronavariant is zorgwekkender dan gedacht; opnieuw blijkt een lockdown nodig. Terwijl je dit nieuws verwerkt, leg je met je beleidswetenschappelijke projectteam de laatste hand aan de uitwerking van een van de wettelijke gezondheidsdoelen. Terwijl je naar taal zoekt die aansluit bij de huidige machthebbers vraag je je af of deze uitwerking de dominosteen is die leidt tot een veerkrachtige publieke gezondheid en de utopische feedbackmechanismen zoals hierboven beschreven …
